# A novel long non-coding RNA lnc-GNAT1-1 is low expressed in colorectal cancer and acts as a tumor suppressor through regulating RKIP-NF-κB-Snail circuit

**DOI:** 10.1186/s13046-016-0467-z

**Published:** 2016-12-03

**Authors:** Chunxiang Ye, Zhanlong Shen, Bo Wang, Yansen Li, Tao Li, Yang Yang, Kewei Jiang, Yingjiang Ye, Shan Wang

**Affiliations:** 1 Department of Gastroenterological Surgery, Peking University People’s Hospital, Beijing, 100044 People’s Republic of China; 2 Laboratory of Surgical Oncology, Peking University People’s Hospital, Beijing, 100044 People’s Republic of China; 3 Peking University People’s Hospital, No. 11 Xizhimen South Street Xicheng District, Beijing, People’s Republic of China

**Keywords:** Long non-coding RNA, lnc-GNAT1-1, RKIP, NF-κB, Snail

## Abstract

**Background:**

The role of long non-coding RNAs (lncRNAs) in colorectal cancer (CRC) progression has not fully been elucidated. This study was designed to report the identification of a novel lncRNA, lnc-GNAT1-1, and its functional role in CRC progression.

**Methods:**

lncRNA expression profile microarray was performed in three paired primary and liver metastatic tissues of CRC, and a novel lncRNA, lnc-GNAT1-1, was identified to be a potential functional lncRNA. Quantitative real-time PCR was used to detect its expression in CRC tissues, cell lines, and patients’ plasma, cell fractionation was used to evaluate its subcellular location. lnc-GNAT1-1 was knockdown by siRNA or overexpressed by a lentivirus vector, then in vitro an vivo experiments were performed to evaluate its biological role and the underlying mechanisms in CRC.

**Results:**

Expression of lnc-GNAT1-1 was decreased in liver metastasis than the primary tumor, while the later one is lower than the paired normal mucosa. Decreased lnc-GNAT1-1 expression was associated unfavorable clinicopathological features and a poor prognosis of CRC patients. In multivariate analysis, lnc-GNAT1-1 was proved to be an independent prognostic factor. In plasma, lnc-GNAT1-1 was significant decreased in CRC patients than healthy donors, and with the TNM stages advanced, the plasma lnc-GNAT1-1 level decreased; Receiver operating characteristic curve (ROC curve) showed that plasma lnc-GNAT1-1 had a moderate to well diagnostic efficiency for CRC. In vitro experiments showed that knockdown of lnc-GNAT1-1 could inhibit the aggressive phenotypes of CRC cell lines. In vivo study showed that overexpression of lnc-GNAT1-1 could suppress the liver metastasis of CRC cells. Finally, we explored the underlying mechanism of the role lnc-GNAT1-1 plays in CRC, and found a positive correlation between lnc-GNAT1-1 and Raf kinase inhibitor protein (RKIP) expression both in cells and in patients’ tissues. We further found that lnc-GNAT1-1 could regulate the RKIP-NF-κB-Snail circuit in CRC.

**Conclusions:**

We have demonstrated in this study that a novel lncRNA, lnc-GNAT1-1, is low expressed in colorectal cancer tissues and plasma, and acts as a tumor suppressor through regulating RKIP-NF-κB-Snail circuit.

**Electronic supplementary material:**

The online version of this article (doi:10.1186/s13046-016-0467-z) contains supplementary material, which is available to authorized users.

## Background

Colorectal cancer is one of the leading cause of cancer-related death around the world, and causes over a half million deaths every year [1]. Despite considerable progresses have been made over the past decades in the diagnostic approaches and therapeutic strategies, the mortality of CRC is still high, especially for those who suffers a distant metastasis. Thus, it is of great importance for us to understand the biology, genetics and epigenetic alterations in CRC, especially the detailed mechanisms underlying distant metastasis, so as to improve the prognosis of CRC patients.

Long non-coding RNAs (lncRNAs) are greater than 200 nucleotides without protein-coding potential. Recent studies have shown that lncRNAs play a crucial role in diverse biological processes, including alternative splicing, nuclear import, imprinting, cell differentiation and RNA decay [2, 3]. The dysregulation of lncRNAs have also been shown to contribute to the initiation and progression of many types of cancers, including colorectal cancer. Previous studies have proved that long noncoding RNA H19 indicates a poor prognosis of colorectal cancer and promotes tumor growth by recruiting and binding to eIF4A3 [4]; LncRNA MALAT1 promotes tumor growth and metastasis in colorectal cancer through binding to SFPQ and releasing oncogene PTBP2 from SFPQ/PTBP2 complex [5]; Another important lncRNA, HOTAIR, could regulate polycomb-dependent chromatin modification and is associated with poor prognosis in CRC.

To explore the role lncRNAs played in CRC liver metastasis process, we performed lncRNA microarray using the “LncRNA + mRNA Human Gene Expression Microarray V4.0” (CapitalBio Technology, Beijing, China) according to standard protocol in our unpublished study. Three pairs of colorectal cancer and liver metastasis tissues were tested. Among the differentially expressed lncRNAs, lnc-GNAT1-1 was significantly low expressed in the liver metastasis tissues than the primary tumor site. It is a sense-overlapping long non-coding RNA located on Chromosome 3 with a total of 11 transcripts, of which four transcripts of lnc-GNAT1-1 were significantly decreased in the metastatic tumor tissue, which indicated that lnc-GNAT1-1 might have participated in the liver metastasis processes of CRC cancer.

However, to the best of our knowledge, the biological functions and the roles lnc-GNAT1-1 plays in cancer has not been reported previously. Hence, in this study, we sought to determine the expression and the biological function of lnc-GNAT1-1 in CRC, especially its role in liver metastasis. Expression levels of lnc-GNAT1-1 were determined not only in the CRC primary tumor and liver metastasis tissues, but also in the plasma of CRC patients, and its correlations with clinicopathological parameters were analyzed. We further studied the influence of lnc-GNAT1-1 on the aggressive phenotypes of CRC cell lines in vitro and in vivo. The regulatory role of lnc-GNAT1-1 on RKIP were also explored to elucidate the potential mechanisms. We have demonstrated that a novel lncRNA, lnc-GNAT1-1, played a vital role in the progression of CRC.

## Methods

### Patients and samples

Sixty-eight CRC patients who were diagnosed and underwent surgery in Peking University People’s Hospital between 2007 and 2015 were included in this study. Fresh colorectal tumor tissues and matched normal colorectal mucosa tissues were obtained from all the 68 patients. Liver metastatic tumor tissues were obtained from 18 of the 68 patients. The specimens were obtained and immediately frozen in liquid nitrogen and stored at −80 °C until RNA or protein extraction. Plasma samples from another 62 CRC patients were collected just before surgery between 2014 and 2016. None of them received any antineoplastic treatment. Plasma from 37 healthy donors with matched age were collected as controls.

### Cell lines and cell culture

The human CRC cell lines SW480, SW620, HT29, LoVo, HCT116, and RKO cells were purchased from the American Type Culture Collection (Manassas, VA, USA) and subcultured and preserved by our lab. Cells were maintained at 37 °C with 5% CO2 in Dulbecco’s Modified Essential Medium (DMEM) medium supplemented with 10% fetal bovine serum (FBS, Gibco), 100 U/mL penicillin (Sigma-Aldrich, St Louis, MO, USA), and 100 μg/mL streptomycin (Sigma-Aldrich).

### RNA extraction and quantitative real-time PCR

Total RNA from cell lines and tissue samples was extracted using Trizol (Invitrogen) according to the manufacturer’s instructions. For the plasma, the total RNAs were extracted using mirVanaTM PARISTM microRNA extraction kit (ABI) according to the manufacturer’s instructions. For lncRNA quantification, GAPDH was used as internal control, and PrimeScriptTM RT Master Mix (TAKARA) were used for reverse transcription and real-time PCR. The primer sequences were as follows: lnc-GNAT1-1 forward: 5′-ATGTGTCCCCAGGTTCCTGTT-3′, lnc-GNAT1-1 reverse: 5′-CCCCTGAGGACTTGAGTAGC-3′; RKIP forward: 5′-GGAACGGGGAGTGTACCAAG-3′, RKIP reverse: 5′-CCATCTGGTCGTAATCTTGAAGG-3′. GAPDH forward: 5′- GCAAGAGCACAAGAGGAAGA-3′, GAPDH reverse: 5′-ACTGTGAGGAGGGGAGATTC-3′. All reactions were performed in triplicate. The fold change for each gene relative to the control group was calculated using the 2 − ΔΔCt method.

### Western blot analysis

Total protein extracts were separated by Trisglycine polyacrylamide gels and transferred to polyvinylidene fluoride membranes (General Electric Healthcare, Buckinghamshire, UK). Membranes were incubated with primary antibodies followed by horseradish peroxidase-labeled secondary antibody. GAPDH was used as a loading control. The antibodies used in the experiments are shown in Additional file 1: Table S1.

### Subcellular fractionation

The separation of nuclear and cytosolic fractions was carried out using the PARIS Kit (Life Technologies) according to the manufacturer’s instructions. For quantification PCR, GAPDH and U6 were used as used as fractionation indicators. Primer sequence for U6 was: Forward: 5′- CTCGCTTCGGCAGCACA-3′.

### Cell transfection

For in vitro assays, to interfere the expression of lnc-GNAT1-1, siRNA interference sequences targeting Lnc-GNAT1-1 were designed and synthetized (GenePharma, Shanghai, China), and a final concentration of 50nM were used for transient transfection; to overexpress lnc-GNAT1-1, full-length human lnc-GNAT1-1 cDNA was cloned into the pcDNA3.1 expression vector (GenePharma, Shanghai, China). Lipofectamine 3000 (Invitrogen, Carlsbad, CA, USA) was used for transfection according to manufacturer’s instructions.

For in vivo studies, lnc-GNAT1-1 overexpression cell line was used. The lnc-GNAT1-1 gene was cloned in to a lentivirus vector LV-GFP-Puro, and RKO cells were used for infection. Stable transfection cells were established by puromycin antibiotic selection applied for 3 days, with a concentration of 5ug/ml. The lnc-GNAT1-1 overexpression cells and control cells were named RKO-LV-lnc-GNAT1-1 and RKO-LV-NC, respectively.

### Cell proliferation assays and flow cytometry analysis

Cell proliferation assay was performed with Cell Counting Kit-8 (CCK8) according to the manufacturer’s protocol, and detected at 0, 24, 48, 72 and 96 h. Cells in each group were tested for 5 replicates.

For colony formation assay, transfected cells were seeded into each well of a 6-well plate on day 0 then incubated for another 12d. Then, the wells were fixed with 4% paraformaldehyde, and stained with 0.1% crystal violet. The form colonies were counted and analyzed by Image J software.

Cells were cultured in 6-wells plate until the cell density reached more than 95% confluence. Then, a vertical wound was scratched using a 100ul microtip. The cells were washed with phosphate-buffered saline (PBS) twice to remove the cell debris. Images were captured at 0 and 24 h or 36 h to assess wound closure.

For cell cycle analysis, cells were stained with propidium iodide (PI) solution (BD Cycletest™ Plus DNA Kit) and were analyzed by a fluorescence-activated cell sorter (FACS) 48 h after transfection. For apoptosis analysis, 48 h after transfection, cells were stained with BD FITC Annexin V Apoptosis Detection Kit I followed by FACS analysis according to the manufacturer’s protocol.

### Cell migration and invasion assays

Cell invasion assay was performed using Corning Polycarbonate Membrane Insertin transwell chamber (Product #3422, Corning Costar Corp, Cambridge, MA, USA). After transfection for 24 h, cells (1 × 10^5^) in serum-free media were placed into the upper chamber to do migration assays (without Martrigel) and invasion assays (with Martrigel, Sigma). Media containing 20% FBS was placed in the lower chamber as a chemoattractant. After 48 h incubation, the non-invading cells were removed with cotton swabs. Migrated or invasive cells at the bottom of the membrane were stained with methanol 0.1% crystal violet and images were captured under the microscope. The number of the cells were counted by Image J software.

### Liver metastasis model in nude mice

Stably transfected cells were washed twice and resuspended in 1 × Hank’s buffer at a concentration of 2 × 10^7^ cells/mL. A 50 μL cell suspension was then injected into the inferior pole of the spleen of Balb/c athymic mice under anesthesia. 4 mice were included in each group. Mice were sacrificed 7 weeks after operation, and the spleens and livers were surgically excised. The number and size of formed tumors in the spleen and liver were documented. Moreover, the metastatic lesions were determined by hematoxylin and eosin (HE) staining.

### Statistical analysis

SPSS 16.0 software (SPSS Inc., Chicago, IL, USA) was used to perform the statistical analysis. Data were presented as mean ± SD from at least three separate experiments. Comparison between groups was made using the Student’s unpaired *t*-test (2-tailed). Paired qPCR relative expression results were compared with Wilcoxon Signed Ranks test. The Chi-squared test (*χ* ^2^ test) was used to evaluate the relationship between the clinicopathological features and lnc-GNAT1-1 expression. Kaplan–Meier analysis and Cox regression analysis was used for survival analysis. For all, *P* values of <0.05 were considered statistically significant.

## Results

### lnc-GNAT1-1 is low expressed in CRC tissues

**Fig. 1:**
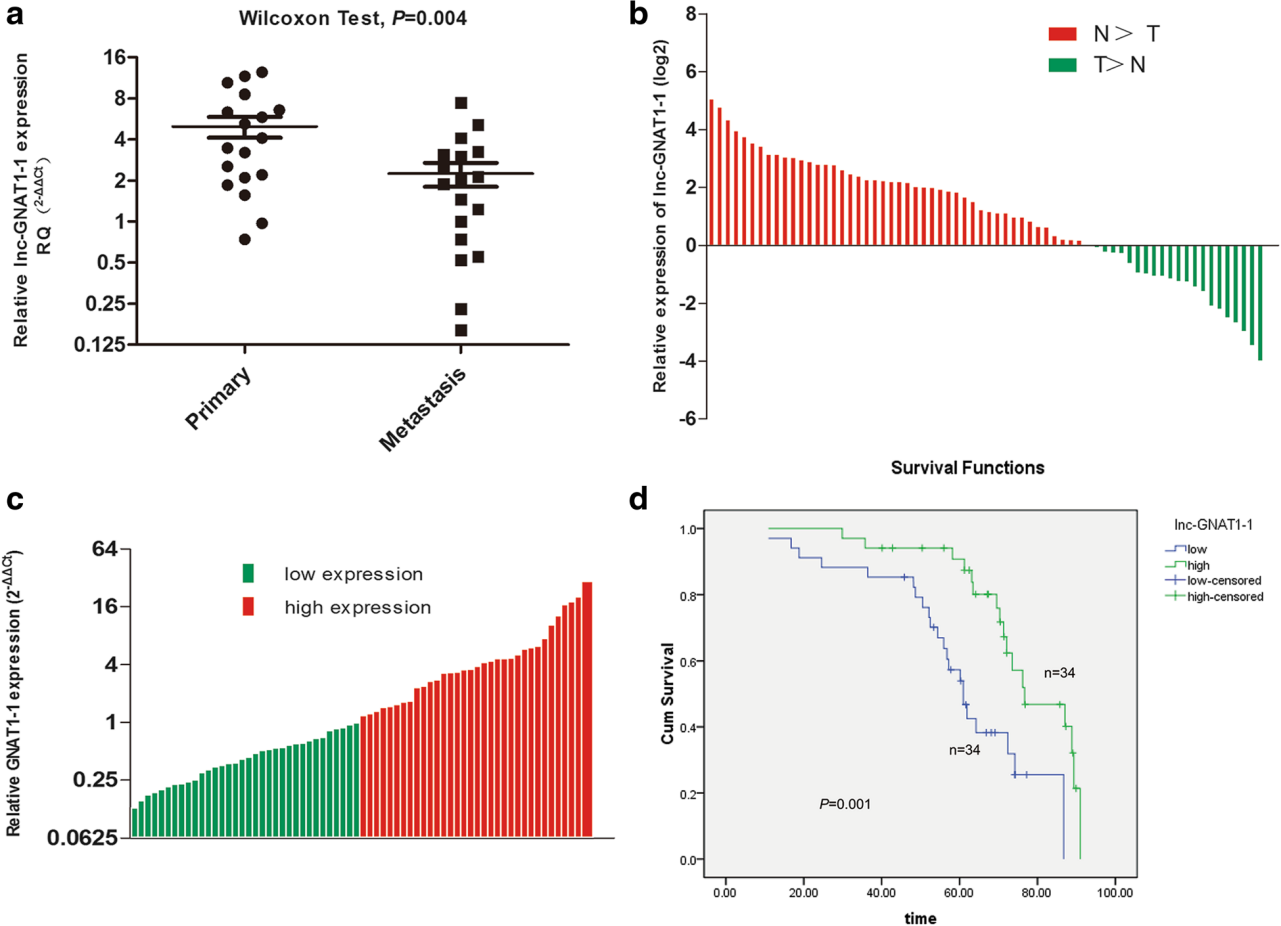
Expression of lnc-GNAT1-1 in CRC tissues and its association with OS. **a** Expression of lnc-GNAT1-1 was decreased in liver metastatic tissues than the primary tumor tissues, with an average fold change of 2.22 (*P* = 0.004). **b** lnc-GNAT1-1 expression was significantly deregulated in tumor tissues compared with the paired adjacent normal tissues, with an average fold change of 2.04 (*P* < 0.001). **c** All the 68 CRC patients were divided into high lnc-GNAT1-1 group (*n* = 34) and low lnc-GNAT1-1 group (*n* = 34) according to the median expression level. **d** Kaplan-Meier analysis showed that low expression of lnc-GNAT1-1 was associated with a poor overall survival of CRC patients (*P* = 0.001)

As mentioned above, we previously conducted lncRNA microarray and explored the global expression profiles of lncRNAs in colorectal cancer primary tissues and liver metastatic tissues. Among the 98 differentially expressed lncRNAs transcripts, we noticed that four transcripts of lncRNA lnc-GNAT1-1 (lnc-GNAT1-1:9, 11, 10, 1) were remarkably higher in primary CRC tissues than the liver metastatic tissues, with an average fold change of 42.81. To verify this result, we determinate the expression of lnc-GNAT1-1 in 18 paired CRC primary and liver metastasis tissues. Results showed that lnc-GNAT1-1 was significantly decreased in liver metastatic tissues compared with primary tumor (Fig. 1a). We further detected lnc-GNAT1-1 expression in another 68 CRC tissues and matched normal mucosa. We found that expression of lnc-GNAT1-1 was significantly up-regulated in tumor tissues. Among the 68 CRC patients, 69.12% (47/68) showed decreased expression of lnc-GNAT1-1 in tumor tissues compared with paired normal mucosa (*P* < 0.001, Fig. 1b). The above data were in agreement with the microarray results and indicated that lnc-GNAT1-1 might be involved in the occurrence and progression of CRC.

### Decreased expression of lnc-GNAT1-1 predicts a poor prognosis of CRC patients

Based on the real-time PCR results of the tissues, we divided all the 68 patients into high expression group and low expression group according to the median expression level (Fig. 1c). As shown in Table 1, the lnc-GNAT1-1 level in cancer tissues was associated with lymphovascular invasion (*p* = 0.012), depth of tumor invasion (*p* = 0.028), distant metastasis (*p* = 0.022), and tumor stage (*p* = 0.027). While no significant correlation was detected with other clinicopathological features, such as age, gender, tumor size, and differentiation.

**Table 1:** Association of lncG expression and clinicopathological features in CRC patients

Clinicopathological features	lncG expression		*P* value
	Low (%)	High (%)	
Gender			
Male	23(56.10)	18(43.90)	0.215
Female	11(40.74)	16(59.26)	
Age			
< 60	8(38.10)	13(61.90)	0.189
≥ 60	26(55.32)	21(44.68)	
Tumor size (cm)			
< 4	14(40.00)	21(60.00)	0.089
≥ 4	20(60.61)	13(39.39)	
Lymphovascular invasion			
Absent	21(41.17)	30(58.82)	0.012
Present	13(76.47)	4(23.53)	
Differentiation			
Well-moderate	24(53.33)	21(46.67)	0.442
Poor	10(43.48)	13(56.52)	
Depth of invasion			
T1 + T2	11(35.48)	20(64.52)	0.028
T3 + T4	23(62.16)	14(37.84)	
Lymph node metastasis			
N0	11(37.93)	18(62.07)	0.086
N1-2	23(58.97)	16(41.03)	
distant metastasis			
M0	22(42.31)	30(57.69)	0.022
M1	12(75.00)	4(25.00)	
TNM stage			
I + II	10(34.48)	19(65.52)	0.027
III + IV	24(61.54)	15(38.46)

**Table 2:** Cox proportional hazard regression model analysis

Variables	Univariate Cox’s regression analysis			Multivariate Cox’s regression analysis		
	Relative risk	*95% CI*	*P* value	Relative risk	*95% CI*	*P* value
Lymphovascular invasion						
present versus absent	3.503	1.692-7.251	0.001			
lncG expression						
high versus low	0.324	0.160-0.656	0.002	0.462	0.216-0.988	0.046
T stage						
T3 + T4 versus T1 + T2	2.633	1.311-5.290	0.007			
N stage						
N1 + N2 versus N0	3.470	1.669-7.213	0.001	2.412	1.083-5.375	0.031
Metastasis						
Yes versus No	5.024	2.436-10.363	<0.001	2.394	1.025-5.596	0.044

The follow-up time of all patients was from March 2007 to November 2015, with a median survival time of 62.50 months. Kaplan-Meier analysis with log-rank test was performed to evaluate the influence of lnc-GNAT1-1 expression on the overall survival (OS) of CRC patients. The patients in the high lnc-GNAT1-1 expression group has a significant longer OS than those in the low expression group (55.75 vs. 68.59 months, *p* = 0.001). In univariate Cox regression analysis of OS, lnc-GNAT1-1 expression, lymphovascular invasion, depth of tumor invasion, lymph node metastasis, distant metastasis and tumor stage were identified as prognostic indicators, while in multivariate Cox regression analysis, only lnc-GNAT1-1 expression (*p* = 0.046), lymph node metastasis (*p* = 0.031) and distant metastasis (*p* = 0.044) could be served as independent prognostic factors (Table 2).

### Plasma lnc-GNAT1-1 is decreased in CRC patients

**Fig. 2:**
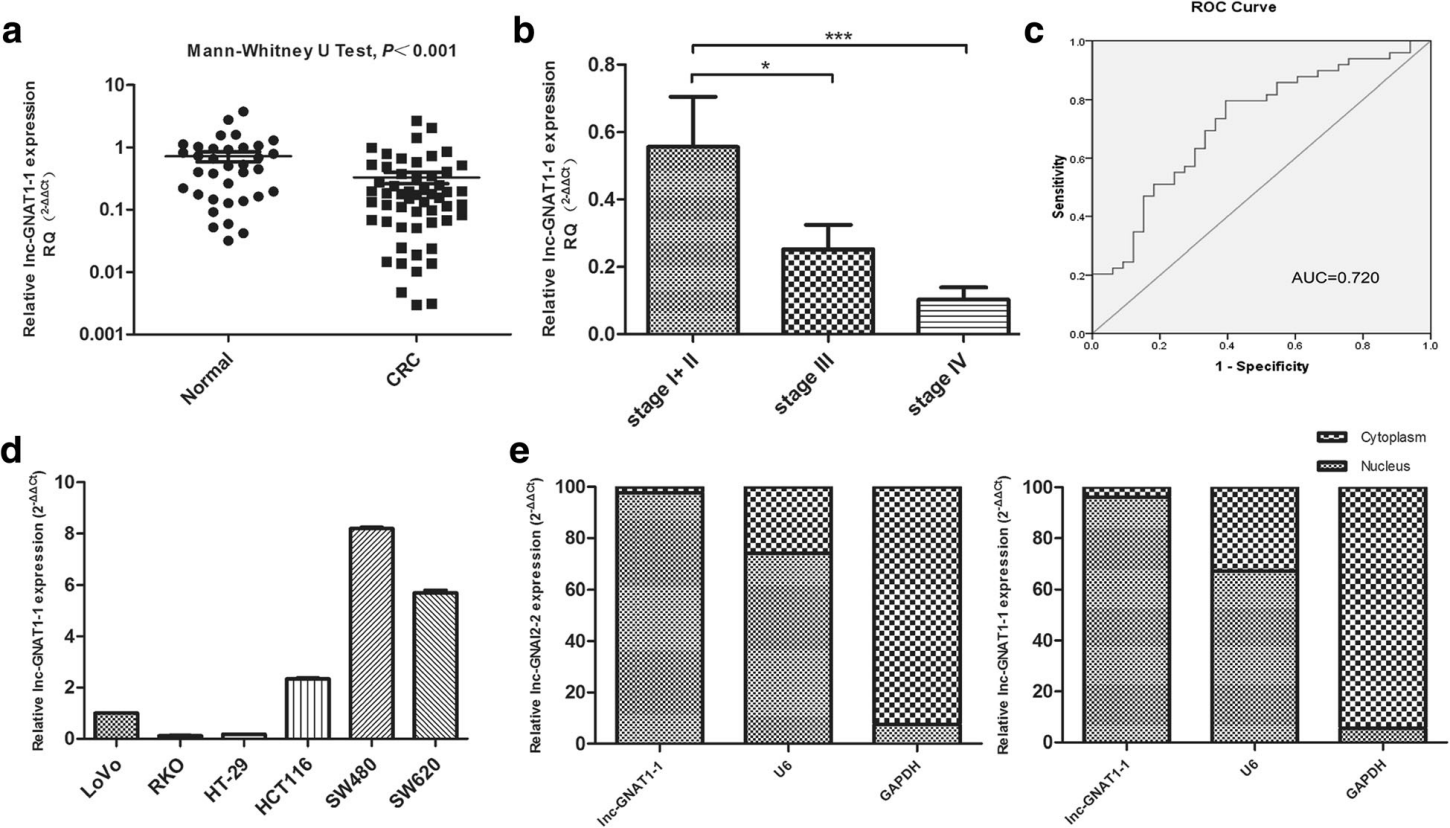
Expression of lnc-GNAT1-1 in CRC patients’ plasma, as well as its expression in CRC cell lines and its subcellular location. **a** Expression of lnc-GNAT1-1 was decreased in plasma of CRC patients than the normal controls (relative expression levels, 0.33 vs 0.72, *P* < 0.001). **b** Plasma lnc-GNAT1-1 levels were decreased with the TNM stages advanced. Its relative expression levels in stage I+ II, stage III and stage IV patients were 0.56, 0.25, 0.10, respectively. **c** ROC curve analysis of lnc-GNAT1-1 for discriminative ability between CRC cases and normal controls, with an AUC of 0.702. **d** Relative expression of lnc-GNAT1-1 in 6 CRC cell lines, with the highest in SW480, and the lowest in RKO cells. **e** Subcellular fractionation for lnc-GNAT1-1 in SW480 and LoVo cells, U6 and GAPDH were set as controls. Lnc-GNAT1-1 has a predominate localization in the nucleus. *P < 0.05, ***P < 0.001

As lncRNAs have the potential to be applied as non-invasive biomarkers for cancer detection, we next detected the level of circulating lnc-GNAT1-1 in CRC patients. Plasma lnc-GNAT1-1 was detected in 88.71% (55/62) CRC patients and 94.59% (35/37) healthy controls. The plasma level of lnc-GNAT1-1 was significantly higher in healthy controls than in CRC patients (*P* < 0.001), with an average fold change of 2.25. Especially, after stratification by TNM stages, plasma lnc-GNAT1-1 level showed a decreased trend with the stages advanced. In our analysis, stage I and stage II cases were grouped together because of limited Stage I cases. The mean relative expression of plasma lnc-GNAT1-1 level in stage I+ II, stage III and stage IV patients were 0.56, 0.25, 0.10, respectively (Fig. 2a), stage III and stage IV patients had significant lower plasma lnc-GNAT1-1 levels than stage I+ II patients. Next, we examined the diagnostic efficiency of lnc-GNAT1-1 in CRC using the ROC curve. As shown in Fig. 2b, the area under the curve (AUC) of the ROC curve was 0.720, which indicated that serum lnc-GNAT1-1 level possessed a moderate to strong diagnostic efficiency for CRC patients.

### Expression and subcellular localization of lnc-GNAT1-1 in CRC cell lines

Next, the expression of lnc-GNAT1-1 was determined in 6 CRC cell lines. As shown in Fig. 2c, lnc-GNAT1-1 had the lowest expression in the poorly differentiated and highly aggressive RKO cells; it had the highest expression in the SW480 cell line, while in its metastatic derivation cell line SW620, the expression level was decreased.

Then, lnc-GNAT1-1 was analyzed for nucleic-cytoplasmic compartmentalization by subcellular fractionation of SW480 and LoVo cells (Fig. 2d). We observed a considerable enrichment of lnc-GNAT1-1 expression in the nucleus versus the cytoplasm, which suggested that lnc-GNAT1-1 was mainly localized in the nucleus and maybe exerted its functions at the transcriptional level or through interacting with the nuclear proteins (Fig. 2e).

### Influence of lnc-GNAT1-1 on the malignant phenotypes of CRC cells

**Fig. 3:**
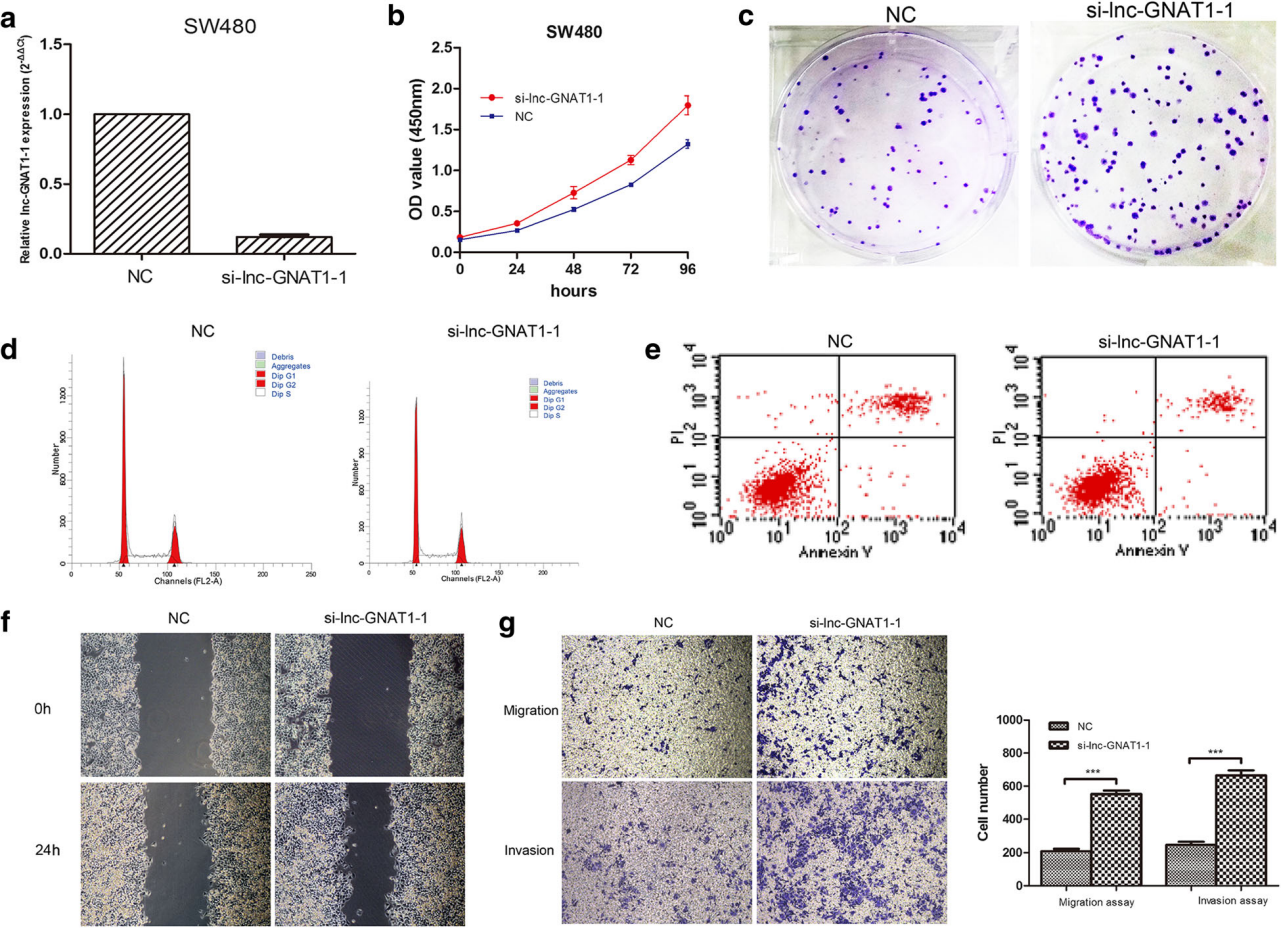
Suppression of lnc-GNAT1-1 promoted proliferation, colony formation, cell motility, migration and invasion of SW480 cells. **a** The efficiency of lnc-GNAT1-1 knockdown was verified by real-time PCR, with the interference efficiency of around 90%. **b** Cell proliferation was determined by CCK8 assay, in which si-lnc-GNAT1-1 cells had a significant promoted proliferation rate than the control cells during a period of 96 h. **c** Plate colony formation assay showed increased colony formation ability of si-lnc-GNAT1-1 cells than the control cells (78.67 ± 4.51 vs. 157.30 ± 10.07, *P* < 0.001). **d** Cell cycle analysis showed that inhibition of lnc-GNAT1-1 could accelerate the cell cycle when compared with the control cells (G0/G1%: 45.50 ± 0.53% vs. 50.65 ± 0.31%, *P* < 0.001). **e** Flow cytometry analysis on apoptosis of cells showed that knockdown of lnc-GNAT1-1 decreased the number of apoptotic cells than control cells (10.17 ± 0.27%, 17.71 ± 0.26%, respectively, *P* < 0.001). **f** Wound healing assay during a period of 24 h showed an increased wound healing capacity of si-lnc-GNAT1-1 cells (Magnification × 100). **g** Transwell migration (*upper*) and invasion (*lower*) assays showed that enhanced migration (551.67 ± 21.83 vs. 208.67 ± 14.57, *P* < 0.001) and invasion (664.67 ± 31.70 vs. 246.00 ± 11.37, *P* < 0.001) capacities of SW480 cells following suppression of lnc-GNAT1-1 (Magnification × 100)

Then, to elucidate the role of lnc-GNAT1-1 in tumor progression, we down-regulated the lnc-GNAT1-1 expression using siRNA or a negative control in SW480 cells. The inhibitory rate (lnc-GNAT1-1 siRNA vs. negative control siRNA) was 88.0% (Fig. 3a). In vitro experiments in SW480 cells showed that inhibition of lnc-GNAT1-1 promoted cell growth in CCK8 cell proliferation assay (Fig. 3b) and increased colony formation ability (Fig. 3c). In flow cytometry analysis, the si-lnc-GNAT1-1 SW480 cells exhibited decreased apoptosis rate (10.16% vs. 17.71%, *P* < 0.001) and accelerated cell cycle (G0/G1%: 45.50% vs. 50.65%, *P* < 0.001) when compared with si-NC cells (Fig. 3d,e). Wound healing ability was also enhanced in the si-lnc-GNAT1-1 cells (Fig. 3f). Finally, in the experiment of transwell migration and invasion assays, inhibition of lnc-GNAT1-1 could significantly enhance the migration and invasion abilities of SW480 cells (Fig. 3g).

**Fig. 4:**
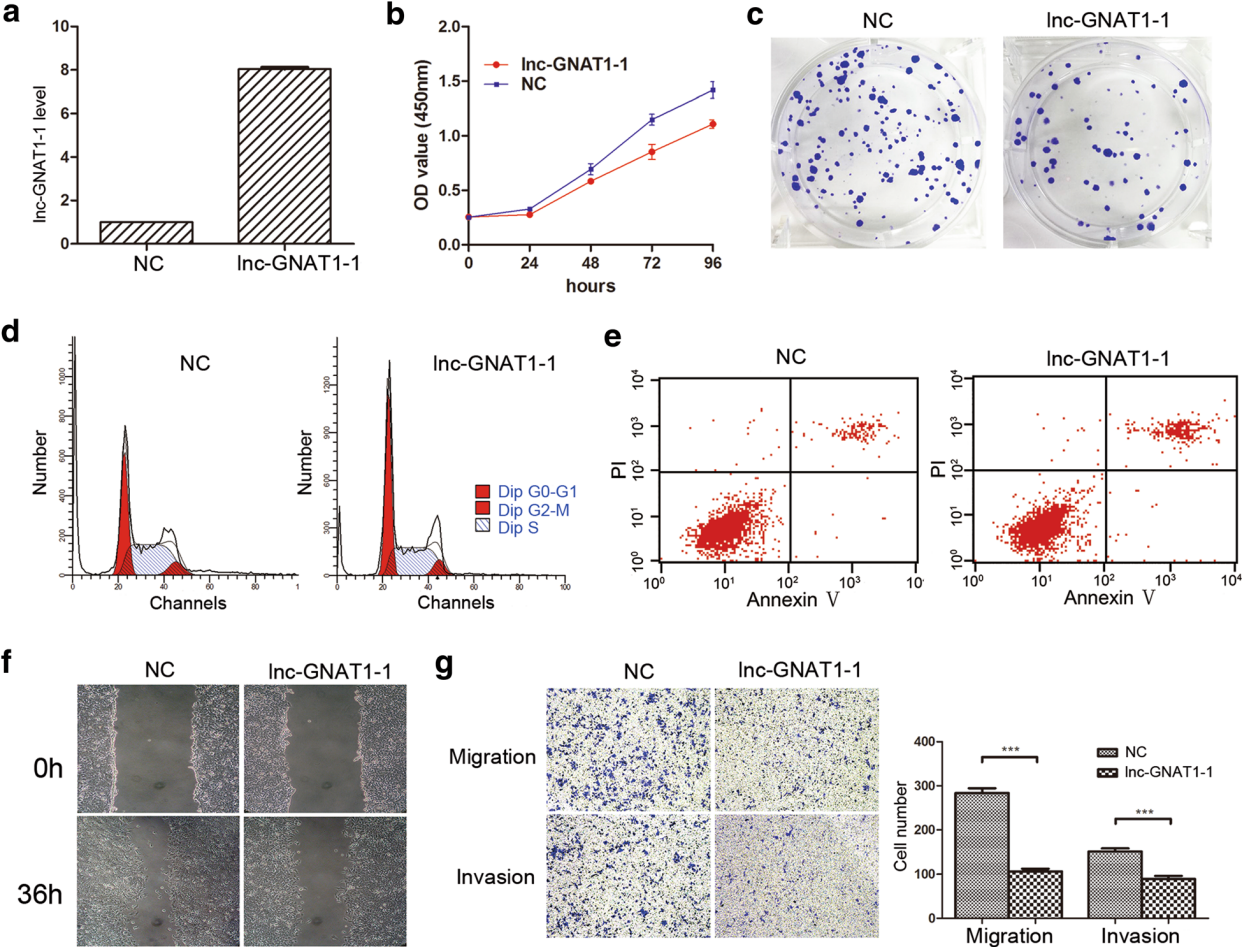
Overexpression of lnc-GNAT1-1 promoted proliferation, colony formation, cell motility, migration and invasion of LoVo cells. **a** LoVo cells were transiently transfected with lnc-GNAT1-1 expression plasmid, and the efficiency of lnc-GNAT1-1 overexpression was verified by real-time PCR, with the fold change of nearly 8 times. **b** CCK8 assay showed that lnc-GNAT1-1 overexpressed cells exhibited a remarkably decreased proliferation rate. **c** Colony formation assay showed that overexpression of lnc-GNAT1-1 decreased the formed colonies of LoVo cells. **d** & **e** Flow cytometry analysis showed that compared with control cells, overexpression of lnc-GNAT1-1 could induce cell apoptosis (13.71 ± 0.12%, 6.96 ± 0.12%, respectively, *P* < 0.001) and hinder cell cycle (G0/G1%: 46.54 ± 0.29% vs. 38.92 ± 0.28%, respectively, *P* < 0.001). **f** Wound healing assay showed that overexpression of lnc-GNAT1-1 could inhibit the mobility of LoVo cell (Magnification × 100). **g** Transwell migration (*upper*) and invasion (*lower*) assays showed that overexpression of lnc-GNAT1-1 could decrease the migration and invasion abilities of LoVo cells (Magnification × 100)

To verify the above results, we further overexpressed lnc-GNAT1-1 in LoVo cells using the pcDNA3.1 plasmid vector. Our results showed that when lnc-GNAT1-1 was overexpressed, the proliferation, colony formation and wound healing abilities of LoVo cells were inhibited (Fig. 4b,c, and f). Flow cytometry analysis also showed an arrested cell cycle (G0/G1%: 46.54% vs. 38.92%, *P* < 0.001, Fig. 4d), as well as a increased cell apoptosis rate (13.71% vs. 6.96%, *P* < 0.001, Fig. 4e) when compared with control cells. In transwell migration and invasion assays, overexpression of lnc-GNAT1-1 attenuated the migratory and invasive abilities of LoVo cells (Fig. 4g). The above in vitro experiments performed in the two CRC cell lines suggested that overexpression of lnc-GNAT1-1 may suppress the malignant phenotypes of CRC cell lines, and inhibition of its expression will bring about more aggressive phenotypes of CRC cells, which was in correspondence with the clinical findings.

### Lnc-GNAT1-1 suppressed the distant metastasis of CRC cell line in vivo

**Fig. 5:**
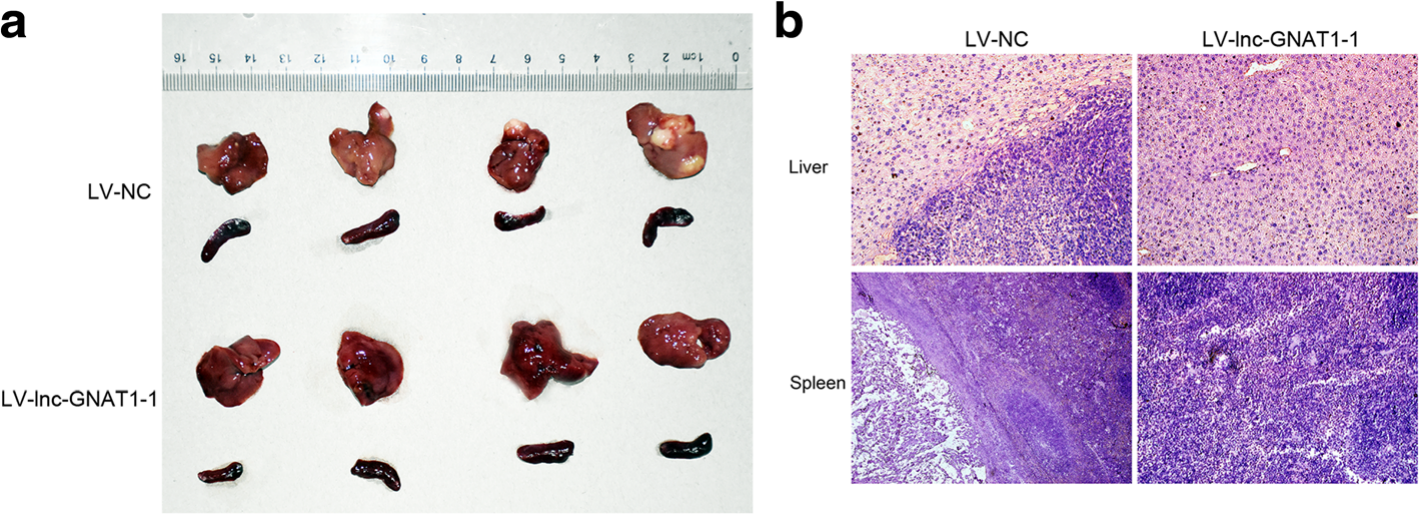
Overexpression of lnc-GNAT1-1 suppressed liver metastasis of CRC cells in vivo*.* **a** Pictures of the excised livers and spleens of BALB/c nude mice. The tumor formation in spleens and metastasis in livers were apparently suppressed in lnc-GNAT1-1 overexpression group (4 in spleen and 4 in liver) than the control group (2 in spleen and only 1 in liver). **b** Representative photos of haematoxylin and eosin (HE) staining sections showed livers and spleens with or without tumors (Magnification × 200)

Next, we evaluated the effect of lnc-GNAT1-1 on CRC liver metastasis process in vivo through injecting lnc-GNAT1-1 overexpression cells (RKO-LV-lnc-GNAT1-1) as well as negative control cells (RKO-LV-NC) into spleens of female nude mice. Seven weeks after injection, the mice were sacrificed, and the formed metastatic tumors on the liver were examined. In LV-lnc-GNAT1-1 group, all four mice formed primary tumors in spleens, as well as metastatic tumors in livers. In contrast, in the control group, only two mice formed tumors in the spleen, and liver metastasis was detected only in one of the above two mice (Fig. 5). The results suggest that overexpression of lnc-GNAT1-1 could significantly suppress liver metastasis of CRC cells.

### Lnc-GNAT1-1 regulates RKIP-NF-κB-Snail circuit in CRC

**Fig. 6:**
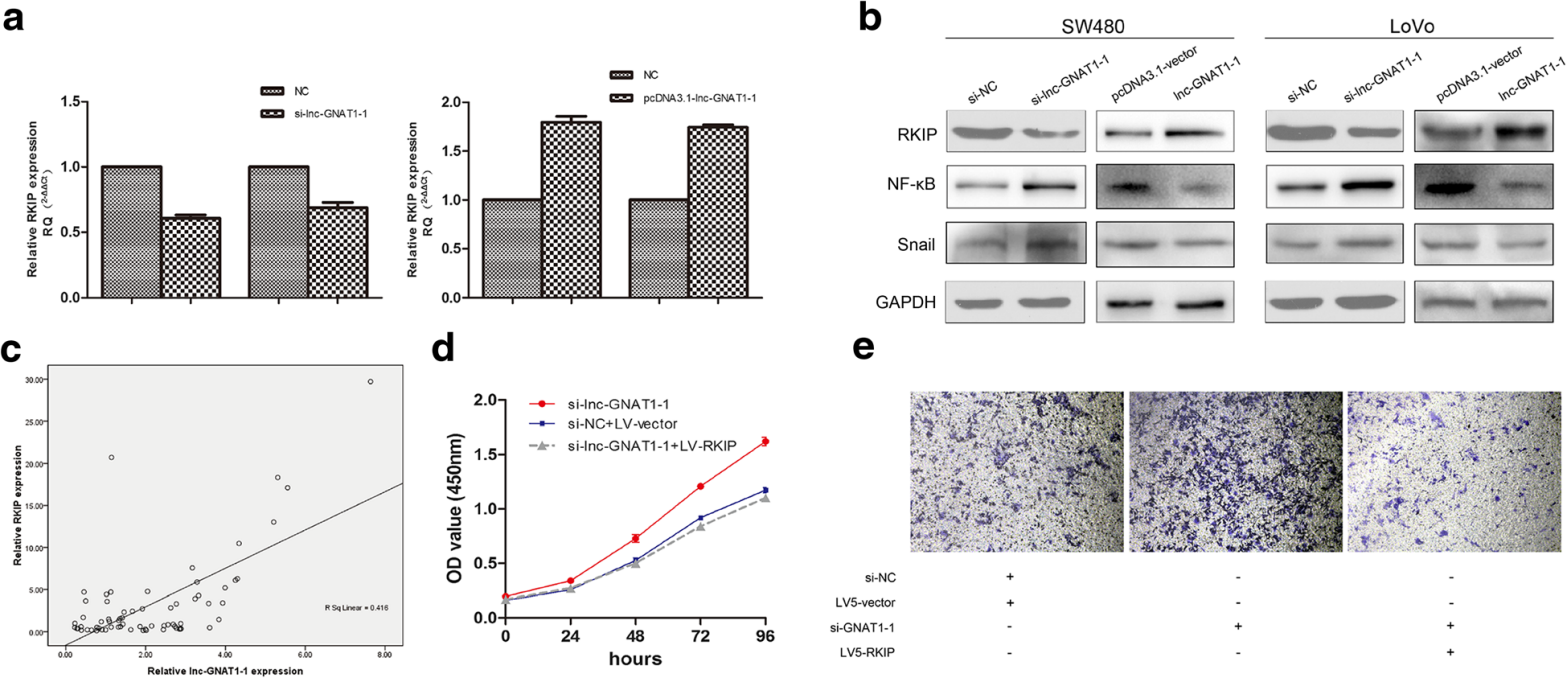
Lnc-GNAT1-1 regulates RKIP-NF-κB-Snail circuit in CRC. **a** Expression of RKIP was decreased or increased following knockdown or overexpression of lnc-GNAT1-1 at the mRNA level. **b** Protein expression levels of RKIP, NF-Kb and Snail were determined in SW480 and LoVo cells following lnc-GNAT1-1 knockdown or overexpression. **c** A positive correlation between expression of lnc-GNAT1-1 and RKIP was detected in 68 CRC patients’ cancerous tissues, with R^2^ = 0.416, *P* < 0.001. **d** Rescue experiment showed that reintroduction of RKIP could attenuate the influence of lnc-GNAT1-1 knockdown on proliferation and (**e)** migration in SW480 cells (Magnification × 100)

We further explored the underlying mechanism of the role lnc-GNAT1-1 played in CRC cancer biology. Based on our microarray data, the mRNA and lncRNA co-expression analysis was performed, and significant correlation was defined as correlation > 0.99 or correlation < −0.99, and *P* value <0.05. A total of 22 genes were identified to be significantly co-expressed with lnc-GNAT1-1, of which, RKIP gene has the highest correlation coefficient (0.9977). Previous studies have shown that RKIP played a tumor suppressor role in cancers [6–8], including CRC [9, 10], through participating in a RKIP-NF-κB-Snail circuitry [11–13]. So in the present study, we decided to investigate whether lnc-GNAT1-1 could regulate the expression of RKIP, and further affect the RKIP -NF-κB-Snail circuitry to play its tumor suppressor role in CRC. We knocked down and overexpressed lnc-GNAT1-1 in SW480 cells and LoVo cells, respectively. Then we detected the mRNA and protein expression levels of RKIP, with results showed that RKIP expression was decreased following lnc-GNAT1-1 knockdown, and vice versa (Fig. 6a and b). We further detected the expressions of NF-κB and Snail proteins in the above cells, with results showed that when lnc-GNAT1-1 was knocked down, expressions of NF-κB and Snail increased, while when lnc-GNAT1-1 was overexpressed, expression of the two proteins decreased (Fig. 6b). sThen, correlation between expression of lnc-GNAT1-1 and mRNA level of RKIP were assessed in the above 68 CRC cancer tissues, and Pearson correlation analysis showed a significant positive correlation between them (*R* = 0.645, *P* < 0.001, Fig. 6c). Furthermore, rescue experiments was performed to see whether the tumor suppressive effect of lnc-GNAT1-1 could be attenuated through reintroduction of RKIP into the cells. CCK8 assay (C. 6d) and transwell migration assay (C. 6e) were performed, and the results had shown a significant rescue effect of RKIP on proliferation and metastasis of SW480 cells with lnc-GNAT1-1 knockdown.

## Discussion

LncRNAs have attracted immense research interests from researchers worldwide in recent years. Based upon our previous lncRNA microarray data, we identified a novel lncRNA, lnc-GNAT1-1, which was significantly low expressed in liver metastatic tissues than the primary CRC tumors. Therefore, we explored the role of lnc-GNAT1-1 in CRC in this study. We found that decreased expression of lnc-GNAT1-1 in cancer tissues was associated with lymphovascular invasion, depth of tumor invasion, distant metastasis, tumor stage, as well as OS of the patients; it could be served as an independent prognostic factor for CRC patients. What’s more, our in vitro and in vivo experiments showed that inhibition of lnc-GNAT1-1 could promote the malignant phenotypes of CRC cells, while overexpression of lnc-GNAT1-1 generate an opposite effect. Thus, our data confirmed the hypothesis that as a novel lncRNA, lnc-GNAT1-1 exerts tumor suppressive activity to inhibit cell proliferation and metastasis in colorectal cancer. The underlying molecular mechanisms might be attributed to its regulation on the RKIP -NF-κB-Snail circuit, which had been demonstrated in this study. Especially, as one of the most investigated transcription factors, NF-κB has been proved to regulate multiple cellular processes in cancer, including proliferation, invasion, metastasis, angiogenesis, and chemoresistance [14, 15].

The role lncRNA played in biology of CRC has been studied in recent years, while studies that focused on CRC liver metastasis were limited. To our knowledge, only one previous study was designed to explore the lncRNAs related with liver metastasis of CRC. Ye and colleagues [16] performed lncRNA arrays to screen for differentially expressed lncRNAs in CRC tissues with synchronous, metachronous, or nonliver metastasis. In their study, 40 differentially expressed lncRNAs that were potentially related to CRC liver metastasis were selected and examined, of which three novel target lncRNAs, termed lncRNA-CLMAT1-3, were verified. The difference between their study and ours is that they compared the differentially expressed lncRNAs in primary tumors, with or without liver metastasis, while our study compared the metastatic tumors with the primary tumors.

Another important finding of this study is that lnc-GNAT1-1 is decreased in plasma of CRC patients than in healthy controls, with an AUC of 0.720 in the ROC analysis, which indicates that lnc-GNAT1-1 has a moderate to well efficiency for the diagnosis of CRC. Recently, many studies have focused on lncRNAs as potential tumor markers for cancer diagnosis and prognosis for their stability and non-invasiveness. Zhou et al. evaluated the potential of 8 lncRNAs as diagnostic markers for gastric cancer, and finally confirmed that plasma H19 could serve as potential diagnostic biomarker for gastric cancer, in particular for early stage patients [17]. In addition, other potential plasma lncRNA markers have been identified, for instance, HULC for hepatocellular carcinoma [18], lncRNA-UCA1 for lung cancer [19], POU3F3 for esophageal squamous cell carcinoma [20]. What’s more, in our study we also found that plasma lnc-GNAT1-1 was related with patients’ TNM stages, that was, with the tumor stages advanced, the plasma lnc-GNAT1-1 level decreased. This suggest that monitoring the plasma lnc-GNAT1-1 level might be help to identify those patients who suffer an advanced stage of disease or distant metastasis early, which will be beneficial for prognosis of the patients.

Although the detailed mechanisms that lncRNAs participate in cancer biology have yet to be elucidated, current studies have shown that the gene regulation role lncRNAs played is mainly through its interacting with proteins, especially nuclear proteins, because lncRNAs (including lnc-GNAT1-1), were predominantly located at the cell nucleus. Previous studies have shown a variety of interactions between lncRNAs and proteins, for instance, LOC389641 with E-cadherin [21], SNHG5 with MTA2 [22], and CASC11 with hnRNP-K [23]. In this study, a significant positive expression correlation between lnc-GNAT1-1 and RKIP was detected. RKIP has been reported to be a tumor suppressors in a variety of cancer types, including colorectal cancer [9, 10, 24], gastric cancer [25, 26], esophageal cancer [27], breast cancer [28], etc., and the mechanisms were mainly attributed to its involvement in the RKIP -NF-κB-Snail circuit in previous reports [13, 29, 30]. In this study, we found a positive correlation between expression of lnc-GNAT1-1 and RKIP, both in cells level and in patients’ tissues. What’s more important, after RKIP was overexpressed, the enhancement of down-regulated lnc-GNAT1-1 on cell proliferation and invasion was hindered, which indicated that the tumor suppressive effect of lnc-GNAT1-1 might be mediated through RKIP. While the detailed regulatory effect between lnc-GNAT1-1 and RKIP was unknown. According to previous reports, RKIP protein was located primarily in the cytoplasm, still it could be found in the nucleus, as indicated by several references [31–33]. So it raises the question about the location that lnc-GNAT1-1 interacts with RKIP, directly in the nucleus or indirectly through some intermediate proteins, such as transcription factors. Actually we present in this study that the other two proteins besides RKIP in the RKIP -NF-κB-Snail circuit could also be regulated by lnc-GNAT1-1. Further work is necessary to determine the details of the interaction between lnc-GNAT1-1 and the RKIP -NF-κB-Snail circuit.

## Conclusion

In conclusion, we have identified a novel lncRNA, lnc-GNAT1-1, which is decreased in CRC tissues and plasma and associated with higher overall survival of CRC patients. It could inhibit the aggressive phenotypes of CRC cells both in vitro and in vivo. We also showed in this study that lnc-GNAT1-1 could regulate the RKIP -NF-κB-Snail circuit in CRC cells. Our results have provide a new therapeutic target for CRC treatment.

## Additional file

Additional file 1: Table S1. Western Blot primary antibodies. (DOCX 13 kb)
