# Supplementary material for: A novel long non-coding RNA lnc-GNAT1-1 is low expressed in colorectal cancer and acts as a tumor suppressor through regulating RKIP-NF-κB-Snail circuit
Source: J Exp Clin Cancer Res. 2016 Dec 3;35:187. doi: 10.1186/s13046-016-0467-z (PMC5135755; doi:10.1186/s13046-016-0467-z)
Supplement: Additional file 1: Table S1. — Western Blot primary antibodies. (DOCX 13 kb) [file 13046_2016_467_MOESM1_ESM.docx]

Additional file 1: Table S1 Western Blot primary antibodies

| Antibody | corporation | Dilution ratio |
| --- | --- | --- |
| Anti-GAPDH | Zhong shan Biotechnology Inc. (TA336621) | 1:2000 |
| Anti-RKIP | Biorbyt (orb162672) | 1:1000 |
| Anti-NF-κB | Cell signaling technology (cst3035) | 1:1000 |
| Anti-Snail | Abcam (ab82846) | 1:500 |
